# Predicting malignant progression in clinically high-risk lesions by DNA ploidy analysis and dysplasia grading

**DOI:** 10.1038/s41598-018-34165-5

**Published:** 2018-10-26

**Authors:** Zuraiza Mohamad Zaini, Helen McParland, Henrik Møller, Kate Husband, Edward W. Odell

**Affiliations:** 1 Head and Neck Pathology, King’s College London, Guy’s Hospital, London, SE1 9RT United Kingdom; 2 Department of Oral and Maxillofacial Clinical Sciences, Faculty of Dentistry, University of Malaya, 50603 Kuala Lumpur, Malaysia; 3 Department of Oral Medicine, Guy’s and St Thomas’ NHS Foundation Trust, Guy’s Hospital, London, SE1 9RT United Kingdom; 4 School of Cancer and Pharmaceutical Sciences, King’s College London, Guy’s Hospital, London, SE1 9RT United Kingdom

## Abstract

The value of image cytometry DNA ploidy analysis and dysplasia grading to predict malignant transformation has been determined in oral lesions considered to be at ‘high’ risk on the basis of clinical information and biopsy result. 10-year follow up data for 259 sequential patients with oral lesions clinically at ‘high’ risk of malignant transformation were matched to cancer registry and local pathology database records of malignant outcomes, ploidy result and histological dysplasia grade. In multivariate analysis (n = 228 patients), 24 developed carcinoma and of these, 14 prior biopsy samples were aneuploid. Aneuploidy was a significant predictor (hazard ratio 7.92; 95% CI 3.45, 18.17) compared with diploidy (*p* < 0.001). The positive predictive value (PPV) for severe dysplasia was 50% (95% CI 31.5, 68.5) and for aneuploid lesions, 33.3% (95% CI 19.0, 47.6). Combined DNA aneuploidy and severe dysplasia increased PPV to 56.3% (95% CI 31.9, 80.6). Diploid-tetraploid and non-dysplastic status had high negative predictive values (NPV) of 94.6% (95% CI 91.4, 97.8) and 99.17% (95% CI 97.4, 100.8) respectively. DNA ploidy predicts malignant transformation well and combining it with dysplasia grading gave the highest predictive value. The predictive values reported here exceed those from other investigations to date.

## Introduction

Squamous carcinoma of the mouth is a relatively rare cancer but one with a poor prognosis and whose treatment carries a high morbidity. One major reason for the high cancer burden is that most patients present at late stage^1^.

A proportion of oral squamous carcinomas arise in pre-existing disorders called the oral potentially malignant disorders (OPMD)^2,3^. OPMD are much more prevalent than carcinomas and identifying them allows patients both surveillance for early diagnosis and treatment to prevent carcinoma developing^4^.

Among OPMD, leukoplakia, erythroplakia^5^ and speckled leukoplakia (white, red and mixed mucosal patches respectively) carry a high risk of malignant transformation and their recognition allows early diagnosis of carcinomas and interventions to prevent malignant transformation. However, the great majority of these oral red and white patches never undergo malignant transformation^4,6^ despite being widely described as ‘high-risk’ and identifying those at sufficient risk to benefit from surgery is insufficiently accurate for clinical practice; many patients are under lifelong review for lesions of no significance and others risk morbidity from repeated biopsy and unnecessary excisions.

The current reference standard test used clinically to predict risk of malignant transformation is the presence and grade of dysplasia seen histologically in a biopsy specimen. However, the grading of dysplasia is subjective and has poor reproducibility^7^ and in several large studies has proved a poor predictor of outcome^8–10^. Dysplasia may regress^11–13^ and non-dysplastic lesions may transform^6,8,14^. Better methods are therefore required.

DNA aneuploidy, a state of having abnormal total nuclear DNA content, is a known hallmark of cancer, drives carcinogenesis and has proven ability to predict development of cancer at several body sites. The predictive value of DNA aneuploidy for malignant transformation in OPMDs has previously been investigated in small case series^15–18^, by association with dysplasia grade^19–23^ and its correlation with other clinical parameters^24,25^.

We have recently proposed that DNA image cytometry (ICM) ploidy analysis is the best predictor of malignant transformation for OPMDs. In a large series of patients with red and white lesions, including those of low and minimal risk representative of referrals from primary care, we showed that DNA ICM ploidy analysis had high positive predictive value for malignant transformation and detected non-dysplastic lesions at risk that would otherwise have been missed using routine methods^26^. This study established the value of DNA ploidy analysis in a population with an overall low incidence and risk of development of oral carcinoma.

The aims of this new study were to establish the predictive value of DNA ploidy analysis in a consecutive series of patients considered to be at higher risk of developing carcinoma, on the basis of specialist clinical evaluation and biopsy. Further differences from previous studies include the use of two different sample preparation methods, analysis using improved software, and a near sequential study population with more complete follow-up data and reduced selection bias.

## Results

### Patient characteristics, samples and follow up

**Figure 1:**
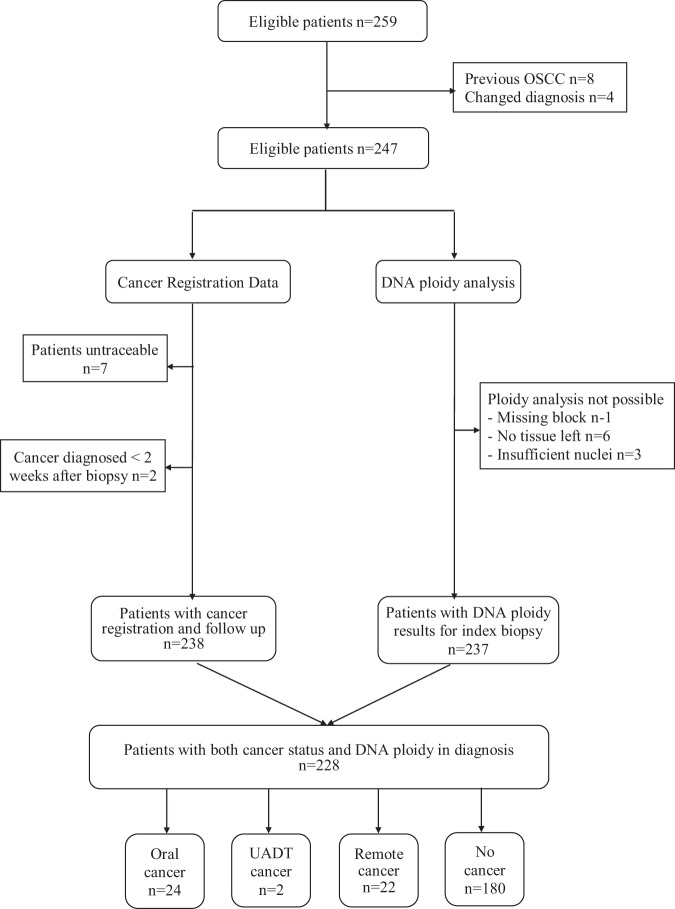
STARD diagram showing recruitment and reasons for exclusion of patients. OSCC = oral squamous cell carcinoma; UADT = upper aerodigestive tract.

From the 259 consecutive patients identified from the archive, 31 were excluded leaving 228 patients in the analysis. Reasons for exclusions and outcomes are shown in a STARD diagram in Fig. 1. Four patients were excluded for other causative factors being identified on review, requiring a change of diagnosis.

The clinical presentations for the study population are shown in Table 1. The majority were white lesions of tongue, buccal mucosa or gingiva. Thirteen patients had other presentations or reasons for biopsy; ulceration in 3 cases, induration in 2, submucous fibrosis in 1. In the remainder, no specific clinical presentation was provided and biopsy was performed for purposes such as monitoring prior dysplasia or to exclude possible carcinoma and no further data was available. One hundred and nine (47.8%) of patients were smokers, 19 (8.3%) ex- smokers and no data was available for 56 (24.6%) patients.

**Table 1:** Clinical descriptors of patient presentations, determined from multiple biopsy specimens when present. *Unknown, see text.

Site	White lesion/keratosis n (%)	Speckled n (%)	Red n (%)	Total n (%)	Total nodular or verrucous n (%)
Tongue	56 (24.6)	4 (1.6)	1 (0.4)	61 (26.8)	3 (1.3)
Buccal mucosa	42 (18.4)	2 (0.9)	3 (1.3)	47 (20.6)	2 (0.9)
Gingiva and/or alveolar ridge	30 (13.2)	1 (0.4)	—	31 (13.6)	2 (0.9)
Soft palate	17 (7.5)	1 (0.4)	—	18 (7.9)	1 (0.4)
Floor of mouth	13 (5.7)	—	1 (0.4)	14 (6.1)	—
Retromolar and fauces	13 (5.7)	1 (0.4)	—	14 (6.1)	—
Multifocal	24 (10.5)	6 (2.6)	—	30 (13.2)	5 (2.2)
Miscellaneous and unknown*	13 (5.7)	—	—	13 (5.7)	
Total	208 (91.2)	15 (6.6)	5 (2.2)	228 (100)	13 (5.7)

The mean age of the patients at time of diagnosis of the index lesion was 55 years (SD 13.9 years) and 122 were male (53.5%) and 106 female (46.5%). The follow up period ranged from 5 to 9.6 years (mean = 7.61, SD = 2.69). Malignant transformation to oral squamous carcinoma occurred in 24 patients, and in 9 of these the carcinoma developed less than 6 months after index specimen. This subgroup was analysed separately. Details of the study population, dysplasia and ploidy diagnosis, subgrouped by time to transformation are shown in Table 2.

**Table 2:** Characteristics of study population, dysplasia grade and DNA ploidy diagnosis.

		Including transformation within 6 months of index lesion		Excluding transformation within 6 months of index lesion	
		n (%)	Malignant transformation n (%)	n (%)	Malignant transformation n (%)
	Total patients	228	24 (10.5)	219	15 (6.9)
Age (years)	Mean (SD)	55 (13.9)	60.4 (10.4)	55 (14.1)	62.2 (11.6)
Gender	Male	122 (53.5)	11 (45.8)	118 (53.9)	7 (46.7)
	Female	106 (46.5)	13 (54.2)	101 (46.1)	8 (53.3)
Dysplasia grade	None	121 (53.1)	1 (4.2)	121 (55.3)	1 (6.7)
	Mild	47 (20.6)	2 (8.3)	47 (21.5)	2 (13.3)
	Moderate	32 (14.0)	7 (29.2)	30 (13.7)	5 (33.3)
	Severe	28 (12.3)	14 (58.3)	21 (9.5)	7 (46.7)
DNA Ploidy	Diploid	183 (80.3)	10 (41.7)	180 (82.2)	7 (46.7)
	Tetraploid	3 (1.3)	0 (0)	3 (1.4)	0 (0)
	Aneuploid	42 (18.4)	14 (58.3)	36 (16.4)	8 (53.3)

Multiple samples were available from 25 patients, from whom further specimens had been taken during the inclusion period of 2004-7; 10 patients had a second biopsy, ten had three biopsies, three patients had four and two had a total of seven biopsies.

### Nuclear extraction method comparison

Ten samples that had sufficient tissues were included for comparison of nuclear extraction methods. Using the newer method, more lymphocyte nuclei were acquired (*p* = 0.01) and fewer fibroblast nuclei were isolated (*p* = 0.03) but numbers of these reference control diploid cells were always far in excess of the number required for analysis (data not shown). There was no difference between the numbers of nuclei excluded as defective. The coefficient of variaton (CV) of the diploid peak in DNA ploidy histograms was better (lower) using the routine method (*p* = 0.007, range 1.34 to 2.57) than the newer method (range 1.72 and 3.75) but all values were well below the 5% value required for diagnosis. There was complete diagnostic agreement between final results obtained with both methods (κ value = 1.00) and samples analysed by both methods were included in the final analysis.

### DNA ploidy and dysplasia

Of the 228 samples, 42 (18.4%) were DNA aneuploid, 3 (1.3%) DNA tetraploid and 183 (80.3%) DNA diploid. DNA aneuploidy was diagnosed mainly in lesions with moderate (36%) and severe (38%) epithelial dysplasia. Almost all non-dysplastic lesions (99.2%) were diploid.

There was a significant association between grade of dysplasia and DNA ploidy status (Pearson *x*^2^ = 73.3, df = 3, *p* < 0.001). Sixteen of 28 cases with severe dysplasia were aneuploid (57.1%) and the remainder were diploid (12/28 = 42.9%). Approximately three quarters of the mildly dysplastic epithelial lesions (74.5%) were diploid. The DNA ploidy diagnoses by subgroup are shown in Table 2 and the association between ploidy diagnosis and dysplasia grade in Table 3.

**Table 3:** Distribution of DNA ploidy diagnosis by degree of dysplasia in all samples.

DNA ploidy	Dysplasia n (%)				Total n (%)
	None	Mild	Moderate	Severe	
Diploid	120 (65.6)	35 (19.1)	16 (8.7)	12 (6.6)	183 (100.0)
Tetraploid	1 (33.3)	1 (33.3)	1 (33.3)	0 (0.0)	3 (100.0)
Aneuploid	0 (0.0)	11 (26.2)	15 (35.7)	16 (38.1)	42 (100.0)
Total n (%)	121 (53.1)	47 (20.6)	32 (14.0)	28 (12.3)	228 (100.0)
**DNA ploidy**	**Dysplasia n (%)**				**Total n (%)**
	**None**	**Mild**	**Moderate**	**Severe**	
Diploid	120 (99.2)	35 (74.5)	16 (50.0)	12 (42.9)	183 (80.26)
Tetraploid	1 (0.8)	1 (2.13)	1 (3.1)	0 (0.0)	3 (1.32)
Aneuploid	0 (0.0)	11 (23.40)	15 (46.9)	16 (57.1)	42 (18.4)
Total n (%)	121 (100.0)	47 (100.0)	32 (100.0)	28 (100.0)	228 (100.0)

Upper table shows number and % of total with each DNA ploidy result and lower table number and % of each dysplasia grade.

### Dysplasia and malignant transformation

Among the 24 (10.5%) of 228 patients who developed carcinoma including those with transformation less than 6 months from index lesion, more than half (58.3%) had severe dysplasia. The mean transformation time was 1.95 years (SD = 2.09). Dysplasia diagnoses by subgroup are shown in Table 2.

**Figure 2:**
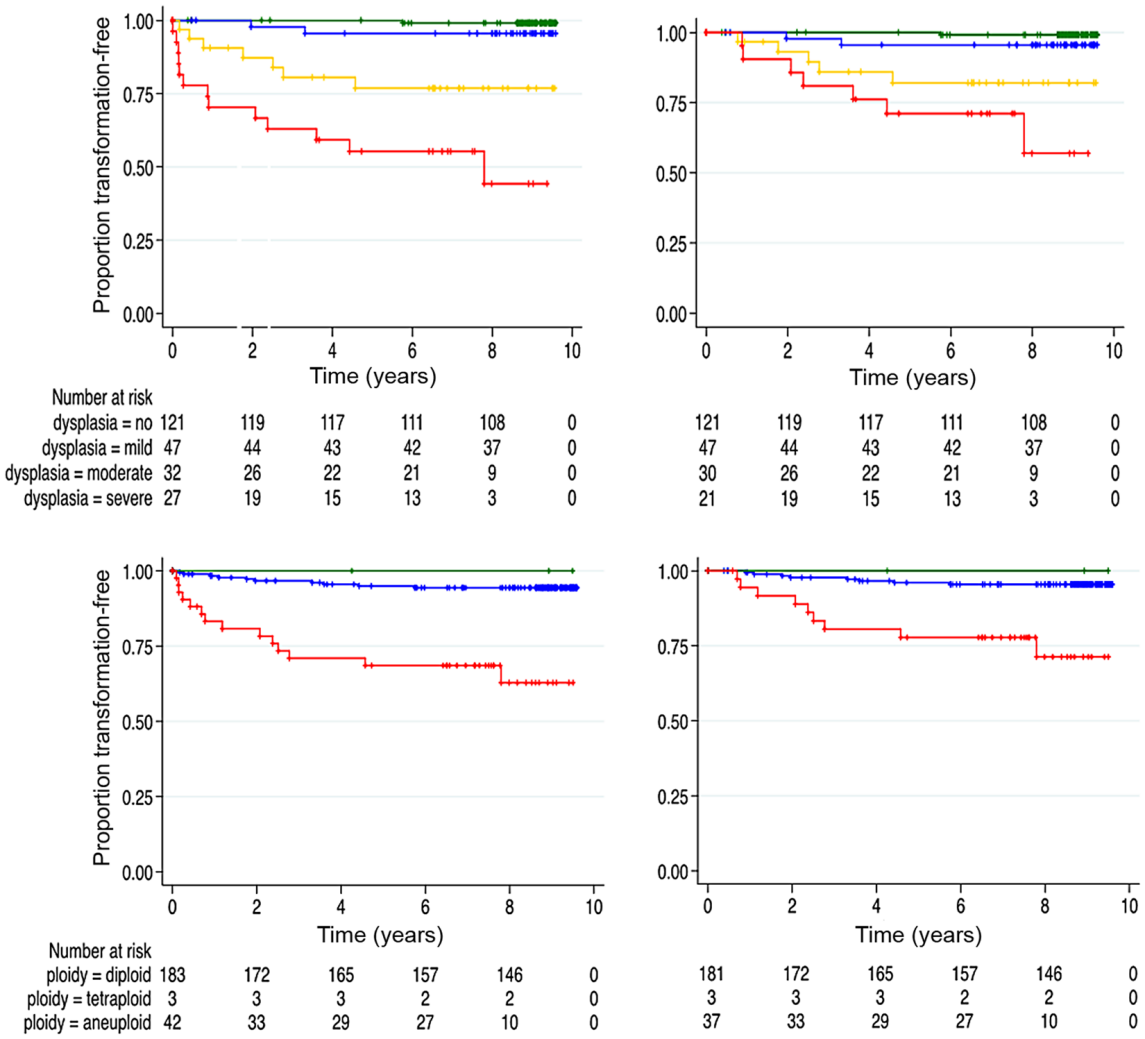
Kaplan Meier curves for malignant transformation. Upper panel. Progression-free proportion by dysplasia grade including (left) and excluding (right) malignant transformation within 6 months of diagnosis of the index lesion (dysplasia grades green, none; blue, mild; amber, moderate; red, severe). Lower panel. Progression-free proportion by DNA ploidy status including (left) and excluding (right) transformation within 6 months of diagnosis of the index lesion (ploidy diagnosis green, diploid; blue, tetraploid; red, aneuploid). The number of patients still at risk at selected time points (x-axis) is shown below each plot. The difference in time to progression for different grades and DNA ploidy status were statistically significant, log rank test *p* ≤ 0.001.

In the one case excluded from Kaplan-Meier and Cox regression analyses because of carcinoma synchronous with the worst dysplasia grade, prior biopsies had been aneuploid 8 months before transformation. Adjusting for age and sex, Kaplan-Meier curves of progression-free estimates in Fig. 2 shows that the time to malignant transformation was significantly shorter in the severely dysplastic group (log rank: *x*^2^ = 76.25, df = 3, *p* < 0.0001).

Hazard ratios were highly significant (*p* < 0.001) indicating the risk of a severely dysplastic lesion developing carcinoma was 81.8 (95% CI 10.5, 635.9) times higher than for a non-dysplastic lesion (Table 4). Multivariate analysis was conducted incorporating all grades of dysplasia and ploidy adjusted for age and gender, and showed that the hazard ratio for severe dysplasia was lower at 54.25 (95% CI 6.6, 448.2) but remained highly significant (*p* < 0.001).

**Table 4:** Cox proportional hazards model on association of dysplasia grade and ploidy status with malignant progression.

		Including transformation within 6 months of index lesion (n = 228)					Excluding transformation within 6 months of index lesion (n = 219)				
		n	Univariate		Multivariate		n	Univariate		Multivariate	
			HR (95% CI)	*P* value	HR (95% CI)	*P* value		HR (95% CI)	*P* value	HR (95% CI)	*P* value
DYSPLASIA	No	121	1.00	—	1.00	—	121	1.00	—	1.00	—
	Mild	47	5.13 (0.46, 56.6)	0.182	3.81 (0.33, 43.96)	0.284	47	5.03 (0.46, 55.7)	0.187	3.90 (0.33, 45.4)	0.278
	Mod	32	29.64 (3.6, 243.6)	0.002	18.78 (2.1, 167.9)	0.009	30	21.57 (2.49, 186.8)	0.005	14.11 (1.45, 137.3)	0.023
	Sev	28	81.82 (10.5, 635.9)	0.000	54.25 (6.6, 448.2)	0.000	21	45.79 (5.52, 379.6)	0.000	30.56 (3.37, 277.4)	0.002
DNA PLOIDY	Dip	183	1.00	—	1.00	—	180	1.00	—	1.00	—
	An	42	7.92 (3.45, 18.17)	0.000	2.41 (0.89, 6.52)	0.083	36	6.79 (2.56, 17.9)	0.000	2.39 (0.72, 7.98)	0.155

HR: hazard ratio. Univariate: adjusted for age, sex. Multivariate: adjusted for age, sex and mutually adjusted between dysplasia grade and ploidy status. Mod = moderate; Sev = severe; Dip = diploid; An = aneuploid.

Excluding patients with transformation within 6 months of the index biopsy (n = 219), the number of cases that underwent malignant transformation was lower at 15 in a mean time of 3.03 years (SD = 1.96). One patient (6.7%) with a non-dysplastic lesion and 2 (13.3%), 5 (33.3%), 7 (46.7%) patients with mild, moderate and severely dysplastic epithelium respectively had undergone malignant transformation.

Patients with higher grades of dysplasia developed carcinoma more rapidly (log rank: *x*^2^ = 40.28, df = 3, *p* < 0.0001, Fig. 2). Although the hazard ratios for moderate and severe dysplasia decreased to 21.6 (95% CI 2.49, 186.8) and 45.8 (95% CI 5.52, 379.6) respectively when compared to the univariate analysis that included early transformation, they remained highly significant with *p* ≤ 0.005 (Table 4).

Multivariate analysis incorporating all grades of dysplasia and ploidy, adjusted for age and gender showed that the hazard ratio for severe dysplasia was lower at 30.56 (95% CI 3.37, 277.4) when compared to univariate analysis. The *p*-value was slightly higher than in the univariate analysis, however still significant (*p* < 0.05). Multivariate analysis proved impossible for smoking and oral subsite though lack of accurate data, which carries some risk of bias.

### DNA ploidy and malignant transformation

In the complete patient group, including those with malignant transformation within 6 months of the index biopsy, 14 (58.3%) aneuploid and 10 (41.7%) diploid lesions progressed to carcinoma (Table 2) in a mean time of 2.05 years (SD = 2.01). The risk of malignant transformation was 7.92 times higher (95% CI 3.45, 18.17) in aneuploid than diploid lesions (*p* < 0.001) (Table 4). Patients with aneuploid lesions developed cancer more rapidly than those with diploid lesions (log rank: *x*^2^ = 33.15, df = 2, *p* < 0.0001) (Fig. 2).

Excluding patients with transformation within 6 months of the index biopsy (n = 219), 8 (53.3%) patients with aneuploid and 7 (46.7%) diploid lesions underwent malignant transformation (Table 2). The mean transformation time was 3.05 year (SD = 1.93). The hazard ratio for a patient with an aneuploid result was 6.79 (95% CI 2.56, 17.9) and was highly significant (*p* < 0.001) compared to patients with diploid lesions (Table 4). Patients with aneuploid index lesions developed carcinoma in a shorter time than diploid (log rank: *x*^2^ = 17.88, df = 2, *p* = 0.0001) (Fig. 2).

In multivariate analysis, the hazard ratio for patients with aneuploid lesions was 2.41(95% CI 0.89, 6.52) and 2.39 (95% CI 0.72, 7.98) when transformation of less than 6 months was included and excluded in the analysis, respectively (Table 4) and these were not statistically significantly different.

### Annual transformation rate

Annual malignant transformation rates for dysplasia grading and DNA aneuploidy are shown in Table 5. For dysplasia grade, total person-year of follow up was 1733.07 and 1731.64 when transformation within 6 months was included and excluded yielding annual rates of 1.32% and 0.87% respectively. Transformation rates increased with the severity of dysplasia. When transformation within 6 months of index lesion was excluded, the rates were 0.09% for the non-dysplastic lesion, 0.53% for mild, 2.68% moderate and 5.77% for severe dysplasia. Annual malignant transformation rates for ploidy calculated with inclusion and exclusion of malignant transformation within 6 months were 1.38% and 0.87% cases per year respectively. By analyzing cases of dysplasia only, the transformation rate was slightly higher at 3.2% and 2.04% cases per year when early transformation was either included or excluded respectively.

**Table 5:** Dysplasia grade- and DNA ploidy-specific annual transformation rates. Mod = moderate; Sev = severe; Dip = diploid; Tet = tetraploid; An = aneuploid.

		Including transformation within 6 months of index lesion				Excluding transformation within 6 months of index lesion			
		n	Number transformed	Person-years follow up	Annual transformation rate % (95% CI)	n	Number transformed	Person-years follow up	Annual transformation rate % (95% CI)
Dysplasia grade	No	121	1	1045.02	0.09 (0.01, 0.68)	121	1	1045.02	0.09 (0.01, 0.68)
	Mild	47	2	378.85	0.53 (0.13, 2.11)	47	2	378.85	0.53 (0.13, 2.11)
	Mod	32	7	187.08	3.74 (1.78, 7.85)	30	5	186.49	2.68 1.11, 6.44
	Sev	28	13	122.13	10.64 (6.18, 18.33)	21	7	121.29	5.77 (2.75, 12.11)
	All	228	23	1733.07	1.32 (0.88, 1.99)	219	15	1731.64	0.87 (0.52, 1.44)
DNA Ploidy	Dip	183	10	1478.24	0.68 (0.36, 1.26)	180	7	1476.69	0.47 (0.23, 0.99)
	Tet	3	0	22.68	0	3	0	22.68	0
	An	42	14	234.31	5.97 (3.54, 10.1)	36	8	232.55	3.44 (1.72, 6.88)
	All	228	24	1735.23	1.38 (0.93, 2.06)	219	15	1731.93	0.87 (0.52, 1.44)

### Predictive values

The number of patients in the tetraploid group was too small for analysis, therefore predictive values were calculated combining diploid and tetraploid groups, justified on the basis of previous analysis^26^. Results for combinations of dysplasia grade and DNA ploidy status are shown in Table 6.

**Table 6:** Positive and negative predictive value of combined dysplasia grading and DNA ploidy.

			INCLUDING 6 MONTHS INDEX BIOPSY		EXCLUDING 6 MONTHS INDEX BIOPSY	
			PPV % (95 CI)	NPV % (95 CI)	PPV % (95 CI)	NPV % (95 CI)
**Total patients**			N = 228		N = 219	
**DYSPLASIA GRADE**						
None			0.83 (−0.8, 2.4)	99.17 (97.4, 100.8)	0.83 (−0.8, 2.4)	99.17 (97.4, 100.8)
Mild			4.26 (−1.5, 9.9)	95.74 (89.9, 101.5)	4.26 (-1.5, 9.9)	95.74 (89.9, 101.5)
Mod			21.88 (7.5, 36.1)	78.12 (63.8, 92.4)	16.67 (3.4, 30.0)	83.33 (70.0, 96.6)
Sev			50.0 (31.5, 68.5)	50.0 (31.5, 68.5)	33.33 (13.1, 53.5)	66.67 (46.5, 86.9)
**DNA PLOIDY**						
Dip or Tet			5.38 (2.1, 8.5)	94.62 (91.4, 97.8)	3.83 (1.0, 6.6)	96.17 (93.4, 99.0)
An			33.33 (19.0, 47.6)	66.67 (59.9, 73.5)	22.22 (8.6, 35.8)	77.78 (64.2, 91.4)
**COMBINATIONS**						
**Ploidy**	**Operator**	**Dysplasia**				
Dip or Tet	AND	No	—	99.2 (97.6, 100.8)	—	99.2 (97.6, 100.8)
Dip or Tet	AND	No OR Mild	—	98.1 (95.9, 100.2)	—	98.1 (95.9, 100.2)
Dip or Tet	AND	Any grade	—	94.6 (91.4, 97.9)	—	96.2 (93.4, 99.0)
Dip or Tet	OR	No	—	94.6 (91.4, 97.9)	—	96.2 (93.4, 99.0)
Dip or Tet	OR	No OR Mild	—	94.9 (91.9, 98.0)	—	96.4 (93.8, 99.0)
Dip or Tet	OR	Any grade	—	89.5 (85.5, 93.5)	—	93.2 (89.8, 96.5)
An	AND	Sev	56.3 (31.9, 80.6)	—	36.4 (7.9, 64.8)	—
An	AND	Sev OR Mod	45.2 (27.6, 62.7)	—	32.0 (13.7, 50.3)	—
An	AND	Any grade	33.3 (19.1, 47.6)	—	22.2 (8.6, 35.8)	—
An	OR	Sev	35.2 (22.4, 47.9)	—	23.9 (11.6, 36.2)	—
An	OR	Sev OR Mod	29.2 (18.7, 39.7)	—	19.4 (9.5, 29.2)	—
An	OR	Any grade	21.5 (13.7, 29.3)	—	14.3 (7.4, 21.2)	—

PPV = positive predictive value; NPV = negative predictive value. Mod = moderate; Sev = severe; Dip = diploid; Tet = tetraploid; An = aneuploid.

Including malignant transformation less than 6 months from index lesion, the positive predictive value (PPV) for severe dysplasia was 50% (95% CI 31.5, 68.5) and 33.3% (95% CI 19.0, 47.6) for aneuploid lesions. When lesions with both DNA aneuploidy and severe dysplasia were combined, the PPV was increased to 56.3% (95% CI 31.9, 80.6). Diploid or tetraploid and non-dysplastic lesions had high negative predictive values (NPV) of 94.6% (95% CI 91.4, 97.8) and 99.17% (95% CI 97.4, 100.8) respectively.

Excluding cases with transformation within 6 months of index lesion (Table 6), the PPV was reduced to 33.3% (95% CI 13.1, 53.5) and 22.2% (95% CI 8.6, 35.8) for severe dysplasia and DNA aneuploidy respectively. When lesions with both DNA aneuploidy and severe dysplasia were combined, the PPV was slightly increased to 36.4% (95% CI 7.9, 64.8). The NPV of diploid or tetraploid was 96% (95% CI 93.4, 99.0) and 99% (95% CI 97.4, 100.8) for non-dysplastic lesions.

### Site specificity

No meaningful analysis of site specificity was possible. ICD coded site data from cancer registration for these years often lacked laterality. Local pathology databases contained cancer site data for 7 patients. In broad terms, sites of index biopsy appeared to be at the site of cancer development in 5 patients (2 lateral tongue, 2 floor of mouth and 1 buccal). In one patient the index biopsy was from floor of mouth and carcinoma developed on the lateral tongue. In one patient, the cancer was remote from the biopsy site (alveolar ridge in opposing jaw). While the descriptions recorded appear to show cancer developing in the same site as index biopsy in 5 cases, each of these sites covers several cm^2^ and site cannot be matched within a few millimetres. This and the small numbers prevent analysis of site specificity.

## Discussion

Prevention of oral squamous carcinoma in patients with OPMD requires an accurate assessment of the risk of malignant transformation to target treatment appropriately. The standard test of grading dysplasia histologically in a biopsy has limitations. It is moderately effective when changes are severe but in many studies has been shown to have too low a positive predictive value^8,9,27^ so that grade is generally considered a poor predictor of transformation. In this study, the additional value of ploidy analysis over dysplasia grading may appear limited because dysplasia grading has proved a good predictor in our centre. However, in the broader context of the many studies showing poor prediction, we hypothesise that DNA ploidy analysis could prove to be the best predictor in centres where dysplasia grade is poorly correlated with outcome.

DNA ploidy analysis has potential as a more objective technique. Unlike conventional ploidy analysis that determines chromosome number, DNA ploidy analysis measures only total DNA content in individual nuclei. This parameter is frequently abnormal in malignant neoplasms and precancerous conditions as a result of chromosomal instability and missegregation during mitosis and has been used in a variety of clinical settings^28^. Analysis using image-based systems, as in this work, has advantages of being able to identify specific small areas of interest for analysis and the ability to separate DNA measurements from different types of cells accurately to provide an internal control.

Previous studies on DNA ploidy analysis by others have been small series with a case-control design^15,16^ or included leukoplakia only^17,18^. We have previously tested DNA ploidy in a large series of patients with OPMD and conditions with which OPMD are frequently confused clinically^26^. In this relatively low risk population including all red and white lesions raising suspicion of an OPMD referred from primary care, DNA ploidy analysis proved as effective as dysplasia grading, had a high negative predictive value to exclude lesions with no risk and was able to identify risk in lesions that appeared innocuous on dysplasia grading. However, that study had limitations. It was not possible to perform DNA ploidy analysis on every sample, and some selection bias was likely to favour analysis of larger lesion with greater sample size. The study evaluated a hospital referral population with an overall low risk of transformation, but the predictive value might be higher in a population that have been screened clinically and would be at higher risk. The positive predictive value of DNA ploidy analysis would normally be expected to rise with a higher incidence of malignant transformation, but the only comparable study showed a similar positive predictive value and low negative predictive value^16^, possibly because of the high transformation rate in the hospital-based sample.

In the present study we have addressed these various issues. The nuclear separation technique required only half as much tissue as the previous protocol, produced equivalent quality samples as assessed by diploid peak CV, and extracted a larger number of nuclei for more accurate determination of 5c and 9c exceeding rates^29^. This enabled analysis of a more representative near sequential series of patients. Patient inclusion involved excluding as far as possible low risk lesions on the basis of clinical descriptors (including age, site, size, multifocality, colour, nodularity, verrucous morphology and habits when available) and biopsy result. Conditions such as lichen planus and chronic hyperplastic candidosis of no risk or minimal risk that were included in our previous study were excluded. Patients were included after examination by a clinician specialising in OPMD whereas in our previous study they were included after referral from primary care. The current study population is more representative of patients who are retained for follow up in hospital clinics after clinical and pathological assessment.

As in our previous study, we have analysed outcome for dysplasia grades and DNA ploidy diagnosis in subgroups with and without transformation within 6 months of the index lesion. Early transformation is considered to reflect sampling error in biopsy and failure to detect a carcinoma that may already have been present. The knowledge that some test results might indicate this very high risk is valuable clinically, and including this group provides the predictive values of most use to clinical practice. It is not possible to comment on whether these early transforming cases did have concurrent carcinoma, but if so it was not appreciated clinically and did not become evident in 6 months. Conversely, analysis with early transformation excluded provides a better reflection of the natural history of OPMD and analysis of this subgroup reflects provides predictive values useful for longer term surveillance. The distributions of age, gender, prevalence and grades of dysplasia, and presence of aneuploidy are comparable to other similar studies, whether early transformation is included or not^15–18,26^. In this series, carcinomas arising in mild and moderate dysplasia arose in the first 2 years of follow up, while those arising in severe dysplasia continued to develop throughout the 9.6 years study duration, consistent with older dysplasia studies^6,14,30,31^ and our and others more recent analyses^26,32^. It is clear that both dysplasia grading and DNA ploidy abnormality indicate long term risk.

We have confirmed the significant correlation between DNA ploidy status and dysplasia shown in our and other previous reports^15,16,20,21,26^ with approximately 40% of DNA abnormal lesions being severely dysplastic and none of the non-dysplastic lesions were aneuploid. However, as previously, many severely dysplastic lesions are DNA diploid at the threshold diagnostic criteria used. As the positive predictive value of dysplasia exceeds that of aneuploidy alone, severely dysplastic but DNA diploid lesions sometimes transform and this raises the possibility that more sensitive diagnostic criteria or higher definition analysis techniques might identify these extra cases at risk.

It can also be concluded that ICM DNA ploidy has value in identifying high risk lesions from among apparently innocuous low risk cases with only mild dysplasia, because 23% of lesions with mild dysplasia harboured aneuploidy. However, the risk in this population appears lower than in higher grade dysplasia with aneuploidy, or possibly develops over a longer period, as neither of the two lesions with mild dysplasia that transformed were aneuploid. However, numbers are too low to analyse this meaningfully.

When evaluating the value of DNA ploidy analysis against dysplasia grading, it is important to note that most studies have failed to show a good correlation between dysplasia grade and malignant transformation^9^. This and our previous study^26^ show relatively good predictive values for dysplasia grading, based on the original diagnostic dysplasia grade made by consensus of two pathologists views, with a third opinion sought in the event of disagreement, as more recently suggested to be best practice^33^.

We have again shown that each grade remains statistically significantly different, even when DNA ploidy was considered simultaneously and whether or not early transformation was included. Despite results from several other large studies^8,9,27^ and the negative tone of much of the literature, we therefore support dysplasia grading as a strong independent indicator of malignant transformation and this is supported by meta-analysis^4,34^.

This study confirms that DNA ploidy analysis predicts malignant transformation. This agrees with our previous analysis of a more diverse low risk series of lesions^26^ and firmly establishes DNA ploidy analysis as a useful clinical test. Others have found more variable results, one similar study reporting similar findings^18^ while others reported a lower hazard ratio^17^, these differences probably largely accounted for by differences in study population with differing risks of transformation.

It is not possible to determine whether DNA ploidy might have predictive superiority over traditional dysplasia as a single test to predict malignant transformation, partly because the value of dysplasia grading is so contentious. In the present study, where dysplasia grading performed well and transformation rates were calculated on an individual patient basis, the statistical significance of DNA aneuploidy was lost when dysplasia grade was considered simultaneously in multivariate analysis and we suggest that DNA ploidy results should be considered an additional source of useful predictive information to predict outcome and combined with other established parameters.

In this series, severe dysplasia but not moderate dysplasia was a better predictor of malignant transformation than ICM DNA aneuploidy. Both absence of dysplasia and ICM DNA diploid status had an equivalent positive and negative predictive value, whether or not early transformation was included in the analysis. While this suggests no advantage to DNA ploidy, our results for dysplasia grading are considerably better than most published studies and we hypothesise that DNA ploidy analysis would have great potential where grading is less effective. Even in the present study DNA ploidy analysis identified DNA aneuploidy in almost a quarter of lesions that showed only mild dysplasia, and these patients would normally be discharged from hospital care.

In the present work, we have been able to combine results from dysplasia grading and DNA ploidy analysis. The combination of severe dysplasia and ICM DNA aneuploidy has increased predictive value over both aneuploidy and severe dysplasia alone and a similar relationship holds with moderate dysplasia. In contrast, adding diploid or tetraploid to the absence of dysplasia showed no benefit to the negative predictive value. However, one of the most valuable aspects of DNA ploidy analysis remains the ability to exclude risk in low risk lesions with more confidence, enabling patients to be discharged for follow up in primary care, avoiding financial cost, inconvenience and repeated biopsy.

In conclusion, the results of this study provide additional evidence that DNA ploidy analysis is a good predictor of malignant transformation in OPMD when applied to oral lesions assessed as having a risk of transformation on clinical grounds. The results also complement the sparse data supporting the efficacy of dysplasia grading to predict malignant transformation in routine clinical practice. The highest predictive values are produced by combinations of the two techniques and the predictive values reported here exceed those from published studies to date. DNA ploidy analysis has value in patient management, both to detect high risk lesions and confidently identify those with no risk.

## Methods

This work complies with UK guidelines and legislation. The study was approved by the Guy’s Hospital Research Ethics Committee and the use of material and data without individual consent has been specifically approved by the UK Patient Information Advisory Group [reference PIAG 4-09(f)2003].

### Case and sample selection

Pathology reports and original request forms for specimens submitted between 2004 and 2007 were searched by hand from the archive of the Department of Oral Pathology, King’s College London. Inclusion criteria were cases with diagnosis of dysplasia confirmed histopathologically or, if non-dysplastic with a clinical description provided by the clinician either as leukoplakia, erythroplakia, white patch/lesion or red patch/lesion or mixed white and red patch/lesion, and a clinical assessment indicating risk of transformation clinically. Histological diagnosis of dysplasia was made by consensus of two pathologists based on WHO criteria at the time of diagnosis, with more emphasis on architectural features than in the WHO 2005 definition. The dysplasia grading was directly equivalent to the WHO 2017 three grade system.

Exclusion criteria were a histological diagnosis, clinical diagnosis or clinical suspicion of any benign red or white oral lesion including candidosis, frictional keratosis, lichenoid reactions or lichen planus. Three patients with a clinical description including striae were included but these also had plaque type lesions and no evidence of lichenoid changes histologically. Patients with prior history of oral squamous carcinoma, carcinoma in first biopsy or in a second biopsy within 2 weeks were excluded. Follow-up data was acquired from cancer registrations and local pathology database, which showed complete concordance, and causes of death data from the Health and Social Care Information Centre. All oral biopsy specimens for the patient within the time frame were identified for analysis and all request forms and records checked for exclusion criteria.

### Preparation of nuclear monolayers

Paraffin blocks were subgrouped into specimens suitable for routine conventional preparation, or smaller samples more suited to a novel method of monolayer preparation, based on the amount of tissue available.

The conventional procedure was performed as described previously^26^. Briefly, multiple sections 50 µm thick equivalent to at least 50 mm total epithelial length were cut, deparaffinized in xylene, rehydrated in ethanols to cold PBS and then incubated in 2 mls 0.05% protease type XXIV (Sigma) at 37 °C in a high speed shaking water bath at 250 rpm for 90 minutes. Released nuclei were filtered through 60mesh nylon gauze and cytospun (Thermo Shandon Cytospin 4) to disperse a monolayer onto a glass slide.

In the improved method for small samples, sections with only 25 mm epithelial length were deparaffinised and rehydrated in the same way and then washed twice in cold PBS by centrifugation for 10 minutes at 1000 rpm and 2200 rpm^35^. Tissue was digested in 1 ml 0.5 mg/ml protease solution (Bacillus licheniformis type VIII, Sigma) at room temperature on a magnetic stirrer using micro-magnetic stirring bars at 600 rpm for 90 minutes. Released nuclei were filtered and monolayers prepared as in the conventional method. Nuclear monolayers were stained by the Feulgen-Schiff method. Efficiency of nuclear preparation by the two methods was compared on an independent set of 10 samples.

### DNA image cytometry

The DNA content of stained nuclei was measured using a PWS (Ploidy Work Station) Grabber system (Room4, Sussex UK), comprising an automated scanning Zeiss Axioplan II microscope (Zeiss, Oberkochen, Germany) equipped with a 546 nm green barrier filter. Images were captured with a black and white AxioCam MRm digital camera (Zeiss, Oberkochen, Germany) with 40x lens providing a resolution for analysis of 162 nm per pixel.

Images from a maximum of 3000 nuclei were automatically scanned and images classified into epithelial, control (lymphocyte and fibroblast) and debris. The PWS Classifier software (Room 4, Sussex UK) was used to discard cut, overlapped and pyknotic nuclei and create DNA ploidy histograms. If cases yielded less than the 300 epithelial nuclei required for diagnosis, analysis was repeated when possible; otherwise the case was excluded. All galleries were edited and all histograms were diagnosed by ZMZ and EWO.

### DNA ploidy diagnostic criteria

Histograms were classified according to previous criteria^26^. A lesion was classified as DNA diploid if only one 2c peak (G0/G1) formed by epithelial nuclei was present, the number of nuclei at 4c peak (G2) did not exceed 10% of the total number of epithelial nuclei and the number of nuclei with DNA content more than 5c did not exceed 1%. A lesion was defined as DNA tetraploid if a 4c peak (DI 1.9–2.1; 3.8–4.2c) exceeding 10% of the total nuclei was present with no other abnormality. Aneuploid lesions were characterized by the presence of one or more peak(s) containing more than 10% of the epithelial nuclei outside the range of the normal diploid (diploid index (DI) 0.9–1.1) or G2 (DI 1.8–2.2c) peaks or when the number of nuclei with a DNA content above 5c exceeded 1% of the total number of epithelial nuclei. At least 300 epithelial nuclei were assessed and samples with a diploid peak coefficient of variation (CV) greater than 5% were excluded. The diploid peak coefficient of variation (ratio of standard deviation to mean of DNA content for all nuclei in the peak) defines the detection threshold for a ploidy abnormality as narrower peaks (with low CV) are more readily discriminated than broad overlapping peaks. The best achievable CV for this type of material is approximately 1, giving a detection limit 1% of a change in DNA content^28^.

### Statistical analysis

The unit of analysis for malignant transformation was the patient. For univariate and multivariate analysis, when a patient had multiple biopsies or specimens, the earliest diagnosis with the most abnormal result was used as the index lesion for analysis (‘first-worst’ dysplasia and ‘first-worst’ ploidy)^26^. When no dysplasia or DNA ploidy abnormality was found, the earliest lesion was taken as the index lesion. Such analysis provides the most appropriate selection to undertake calculation of annual transformation rates, as opposed to average rates.

A single patient was excluded from the dysplasia analysis because the worst grade of dysplasia was diagnosed at the same time as the carcinoma. However, lower grades of dysplasia and DNA aneuploidy preceded the carcinoma allowing the case to be included in the univariate analysis of DNA ploidy. Patients were grouped for separate analysis into those who developed a carcinoma after and those who developed carcinoma within 6 months of their index lesion.

The endpoint date was defined as one of four outcomes: the date carcinoma developed, the last follow-up date when no progression had occurred, the date of death if the patient had died of oral carcinoma or the date of death from any other cause.

The association between ploidy diagnosis and dysplasia grade was calculated using the Pearson chi-square test. Kaplan-Meier methods were used to estimate the time to progression and percentage of patients who underwent malignant transformation by dysplasia and ploidy diagnosis. Survival curves were compared using the log rank test. Cox regression methods were used to investigate the main independent predictors of malignant transformation. Adjusting for age and gender, each dysplasia grade and ploidy result category was assessed separately in a univariate Cox proportional hazards model. In the multivariate model, adjustment involved age, sex and mutual adjustment between dysplasia grade and ploidy status. Hazard ratios (HR) with 95% confidence interval (95% CI) and *p* values were reported and *p* < 0.05 was considered statistically significant. Positive and negative predictive values, sensitivity and specificity were calculated from 2 × 2 tables with malignant transformation as reference (‘gold standard’). Annual malignant transformation rates were calculated based on actual person-years of follow up in order to take into account the time to transformation.

Comparison between conventional and novel methods for nuclear extraction was made using the Wilcoxon signed rank test. The reproducibility of DNA ploidy diagnosis was evaluated by using Cohen’s kappa coefficient. *P*-values < 0.05 were regarded as statistically significant.
